# Sexual Dimorphism in the Regulation of Estrogen, Progesterone, and Androgen Receptors by Sex Steroids in the Rat Airway Smooth Muscle Cells

**DOI:** 10.1155/2016/8423192

**Published:** 2016-03-24

**Authors:** Abraham Zarazúa, Aliesha González-Arenas, Gabriela Ramírez-Vélez, Blanca Bazán-Perkins, Christian Guerra-Araiza, María G. Campos-Lara

**Affiliations:** ^1^Unidad de Investigación Médica en Farmacología, Hospital de Especialidades, Centro Médico Nacional Siglo XXI, 06725 Ciudad de México, Mexico; ^2^Departamento de Medicina Genómica y Toxicología Ambiental, Instituto de Investigaciones Biomédicas, Universidad Nacional Autónoma de México, Ciudad Universitaria, 04510 Ciudad de México, Mexico; ^3^Facultad de Ciencias Químicas de la Universidad La Salle, 06140 Ciudad de México, Mexico; ^4^Departamento de Hiperreactividad Bronquial, Instituto Nacional de Enfermedades Respiratorias Ismael Cosío Villegas, 14080 Ciudad de México, Mexico; ^5^Hospital Infantil de México Federico Gómez, 06720 Ciudad de México, Mexico

## Abstract

The role of sex hormones in lung is known. The three main sex steroid receptors, estrogen, progesterone, and androgen, have not been sufficiently studied in airway smooth muscle cells (ASMC), and the sex hormone regulation on these receptors is unknown. We examined the presence and regulation of sex hormone receptors in female and male rat ASMC by Western blotting and flow cytometry. Gonadectomized rats were treated with 17*β*-estradiol, progesterone, 17*β*-estradiol + progesterone, or testosterone. ASMC were enzymatically isolated from tracheas and bronchi. The experiments were performed with double staining flow cytometry (anti-*α*-actin smooth muscle and antibodies to each hormone receptor). ER*α*, ER*β*, tPR, and AR were detected in females or males. ER*α* was upregulated by E2 and T and downregulated by P4 in females; in males, ER*α* was downregulated by P4, E + P, and T. ER*β* was downregulated by each treatment in females, and only by E + P and T in males. tPR was downregulated by P4, E + P, and T in females. No hormonal regulation was observed in male receptors. AR was downregulated in males treated with E + P and T. We have shown the occurrence of sex hormone receptors in ASMC and their regulation by the sex hormones in female and male rats.

## 1. Introduction

Steroid sex hormones, that is, estradiol (E2), progesterone (P4), and testosterone (T), play an active role in different pulmonary processes. For instance, female premature neonates have more developed lungs compared with male neonates, a fact contributing to a higher incidence of respiratory distress syndrome in male children [1]. During prepubertal childhood female lungs are smaller as compared to males [2]. Moreover, the decrease of PC_20_FEV_1.0_ (provocative concentration causing a 20% fall in the forced expiratory volume in 1 s after maximal inhalation) in women with perimenstrual asthma has been associated with an increase in sputum testosterone levels [3]. Sex differences are also related to respiratory functions. In human airway epithelial cells, 17-*β* estradiol stimulates endothelial nitric oxide synthase activity in a manner dependent on calcium influx; such activity is completely inhibited by the estrogen receptor antagonist ICI 182,780 [4]. Likewise, idiopathic pulmonary fibrosis is more prevalent in men than in women [5, 6]. All these data suggest that sex hormones play a role in the modulation of airway physiology.

One of the main structural elements of the airways is the smooth muscle layer that is key to regulation of airway caliber and tone. Since epidemiological and preclinical data suggest sex differences in asthma, and the potential effects of estrogens on airway function are foreseen, there has been increasing interest in sex steroid regulation on airway smooth muscle function. Sex hormones affect airway smooth muscle (ASM) function as they modulate the presence and/or levels of some agonist receptors. Following lipopolysaccharide (LPS) administration, male mice developed greater airway hyperresponsiveness than females, which was in turn decreased in castrated males and increased in females which were treated with exogenous testosterone. Such sex differences are likely mediated by androgens [7]. Additionally, progesterone and its 5*β*-pregnanolone metabolites prevent the ASM contraction induced by histamine, carbachol, or calcium [8]. On the other hand, an* in vitro* study in bovine airway smooth muscle strips suggests that testosterone contributes to relaxation [9].

Some of the actions of P4, E2, and T are mediated by their nuclear receptors: progesterone receptors (PR), estrogen receptors (ER), and androgen receptors (AR), respectively. These receptors are members of the nuclear receptor family that activate transcription of different genes. Both PR isoforms (PR-A and PR-B) have been found in homogenates of the rat lung, and the predominant isoform is PR-A [10, 11]. ER isoforms have also been detected in the lung; ER-*β* is the predominant isoform detected in this tissue [12–14]. Rat lung cytosolic fractions display binding activities for androgenic steroids [13]. Differences in ER and PR receptor lung content during the estrous cycle and between males and females have been reported [11]. In female rats, cyclic changes in AR lung binding activities corresponding were evident during the estrous cycle [13].

Both messenger RNA and protein of ER-*α* have been found in human bronchiolar and freshly obtained bronchial epithelial cells from sheep [4]. The expression of ER-*β* (mRNA and protein) in human airway smooth muscle and epithelial cells has also been observed [15]. The presence of AR has been shown in epithelial, mesenchymal, and smooth muscle cells surrounding arteries of developing lung from male and female mice [16]. When ER (*α* and *β*) were examined in lung tissue, both receptors were found to be localized primarily in bronchial and bronchioloalveolar epithelium: ER-*α* was detected mainly in epithelial cells cytoplasm, whereas ER-*β* was detected in the nucleus [17]. Vegeto et al. [18] observed in mice with lung inflammation that ER-*α* was expressed in lung resident and infiltrated inflammatory cells and that E2 treatment reduced inflammation [18]. All this evidence indicates the key participation of steroid receptors in the inflammatory process of the respiratory tree.

Despite the evidence indirectly pointing out the presence of steroid hormone receptors in rat lung tissue, studies assessing the presence of sex hormone receptors in ASMC have been focused on estrogen receptors. The aim of this study was to investigate their regulation by sex steroid hormones of the levels of estrogen, progesterone, and androgen receptors in airway smooth muscle cells from male and female rats.

## 2. Materials and Methods

### 2.1. Animals

Adult male (240–260 g) and female (190–210 g) Sprague-Dawley rats were obtained from Servicio de Cirugía Experimental y Bioterio of the Centro Médico Nacional Siglo XXI, Instituto Mexicano del Seguro Social. Animals were maintained under a 12:12 h light/dark cycle with food and water available* ad libitum*. Animals were gonadectomized under ketamine (90 mg/kg Anesket 1000 mg/10 mL, Pisa Agropecuaria, Atitalaquia, Hidalgo, Mexico) and xylazine (10 mg/kg Procin, Pisa Agropecuaria, Atitalaquia, Hidalgo, Mexico) anesthesia. Two weeks later rats were randomly assigned to control and hormonal treatment groups. Animals were treated in accordance with the recommendations of the Guide for the Care and Use of Laboratory Animals [19]. Special care was taken to minimize animal suffering and to reduce the number of animals used to the necessary minimum.

### 2.2. Hormonal Treatments

The control and hormonal treatment groups were as follows: (1) vehicle (corn oil, Jabón la Corona, Ecatepec, Estado de Mexico, Mexico) in the same volume as the hormonal treatments, (2) 17*β*-estradiol 3-benzoate (E2) (Sigma-Chemical Co., St. Louis, Missouri, USA) 0.05 mg/Kg/two consecutive days, (3) progesterone (P4) (Sigma-Chemical Co., St. Louis, Missouri, USA) 4 mg/Kg/one day, (4) E2 two days followed by P4 on the third day (E2 + P4) (in the above mentioned doses), and (5) testosterone propionate (T) (Fluka, Sigma-Aldrich, St. Louis, Missouri, USA) 1 mg/Kg/three consecutive days. Twenty-four hours after the hormone treatment rats were killed by a sodium pentobarbital overdose (200 mg/kg) (Sedalphorte, Salud y Bienestar Animal, Mexico City, Mexico).

### 2.3. Airway Smooth Muscle Cells Isolation

Trachea and lungs were removed and placed in Krebs-Ringer bicarbonate (RKb) solution (mM): 120 NaCl (Sigma-Aldrich Inc., St. Louis, Missouri, USA), 4.7 KCl (Sigma-Aldrich Inc., St. Louis, Missouri, USA), 1.2 MgSO_4_ (Caledon Laboratories LTD, Georgetown, Canada), 1.2 KH_2_PO_4_ (Técnica Química S.A., Mexico), 25 NaHCO_3_ (Caledon Laboratories LTD, Georgetown, Canada), 2.5 CaCl_2_ (Sigma-Aldrich Inc., St. Louis, Missouri, USA), and 11 glucose (Sigma-Aldrich Inc., St. Louis, Missouri, USA), maintained at 37°C and bubbled with a mixture of 95% O_2_–5% CO_2_, pH = 7.4. The lung tissue was carefully removed to expose bronchi. Connective tissue was removed from trachea and bronchi, and then airway smooth muscle was isolated and placed in a solution containing the following (mM): 137 NaCl, 5 KCl, 1.1 CaCl_2_, 20 NaHCO_3_, 1 KH_2_PO_4_, 11 glucose, 25 HEPES (Sigma-Aldrich Inc., St. Louis, Missouri, USA), 1.5% collagenase (Sigma-Aldrich Inc., St. Louis, Missouri, USA), and 0.8 IU elastase type IV (Sigma-Aldrich Inc., St. Louis, Missouri, USA), pH = 7.4. Airway smooth muscle was incubated at 37°C and dissociated by pipetting up and down three or four times in an hour. Airway smooth muscle cells (ASMC) were strained from complete tissue pieces and centrifuged (3500 rpm at 4°C for 5 min).

### 2.4. Protein Extraction and Western Blotting

In order to detect the protein levels of steroid hormone receptors, ASMC were lysed in a buffer consisting of TRIS-HCl (Bio-Rad Laboratories, Hercules, CA) 10 mM, EDTA (Research Organics Inc., Cleveland, OH) 1 mM, NaCl (J. T. Baker, Xalostoc, Mexico) 150 mM, 1% Igepal CA-630 (Sigma-Aldrich, Inc., St. Louis, MO), and protease inhibitor cocktail set VII, containing 4-(2-aminoethyl) benzenesulfonyl fluoride hydrochloride (AEBSF), bestatin, E-64, pepstatin A, and phosphoramidon (Calbiochem, EMD Biosciences, Inc. La Jolla, CA). Total cell proteins were obtained from the supernatants after centrifugation of cell lysates at 12,000 rpm at 4°C for 15 min, run (20 *μ*g) on 12% sodium dodecyl sulfate-polyacrylamide gel electrophoresis (SDS-PAGE), and transferred polyvinylidene difluoride (PVDF) membranes (RPN303D, GE Healthcare, Buckinghamshire, UK). Membranes were blocked with 5% skim milk/0.05% Tween 20 (P1379, Sigma-Aldrich Inc., St. Louis, Missouri, USA) in PBS pH = 7.4 for 30 min at room temperature and then incubated overnight with 1 : 1,000 anti-steroid hormone receptor (SHR) at 4°C, anti-ER*α* (sc-542, Santa Cruz Biotechnology, San Cruz California, USA), anti-ER*β* (sc-6821, Santa Cruz Biotechnology, San Cruz California, USA), anti-tPR (total progesterone receptor) (ab2764, Abcam, Cambridge, Massachusetts, USA), and anti-AR (ab74272, Abcam, Cambridge, Massachusetts, USA). Depending on primary antibodies, blots were incubated for 1 h with a 1 : 10,000 dilution of horseradish peroxidase-labeled donkey anti-goat, goat anti-mouse, or goat anti-rabbit IgG (sc-2033, sc_2005, sc-2004, Santa Cruz Biotechnology, San Cruz California, USA) and detected by enhanced chemiluminescence plus substrate system (ECL, Amersham ECL Western Blotting Systems, GE Healthcare, Buckinghamshire, UK) in Kodak dental films (Eastman Kodak Company, Rochester, New York, USA).

To adjust for possible differences in total protein loaded to each lane, blots were stripped overnight with glycine (Bio-Rad Laboratories, Hercules CA) (0.1 M, pH 2.5, 0.5% SDS) at 4°C and reprobed with 1 : 1,000 goat anti-glyceraldehyde-3-phosphate dehydrogenase (GAPDH) (sc-166545, Santa Cruz Biotechnology, San Cruz California, USA) over night at 4°C. Blots were incubated for 1 h with 1 : 10,000 HRP-labeled donkey anti-goat IgG and detected by ECL. Levels of all proteins analyzed were normalized to those of GAPDH and examined by densitometry as described in the software manual.

### 2.5. Densitometry

Briefly, bands were measured by means of the Kodak 1D Image Analysis Software Windows version 3.5 software using background correction (Eastman Kodak Company, Rochester, NY, USA). The area to be measured was manually adjusted to include the majority of each band without any background. Net intensities in Kodak light units were used to calculate the ratios of problem protein/GAPDH. Values obtained for each band were corrected for the mean of all bands of the same protein divided by its correspondent GAPDH value calculated in the same manner and results represent the data obtained from such calculations.

### 2.6. Flow Cytometry

To be able to determine the levels of SHR exclusively in smooth muscle cells, we performed a double staining of the cells using alpha actin smooth muscle and the steroid hormone receptor that was being analyzed at the moment. ASMC were incubated with 200 *μ*L, 1x Perm/Wash buffer (BD Biosciences, San Diego, California, USA) for 20 minutes at 4°C. After that, ASMC were incubated separately with anti-SHR antibodies (1 : 1,000 anti-ER*α* (sc-542, Santa Cruz Biotechnology, San Cruz California, USA), anti-ER*β* (sc-6821, Santa Cruz Biotechnology, San Cruz California, USA), anti-tPR (ab2764, Abcam, Cambridge, Massachusetts, USA), and anti-AR (ab74272, Abcam, Cambridge, Massachusetts, USA)) for 20 minutes at 4°C. Then, ASMC were incubated with Alexa Fluor 647chicken anti-specific IgG (A21443 Alexa Fluor 647 chicken anti-rabbit IgG, A21463 Alexa Fluor 647 chicken anti-mouse IgG, A21469 Alexa Fluor 647 chicken anti-goat IgG, Invitrogen, Carlsbad, California, USA) for 20 minutes at 4°C. Finally, ASMC were incubated with 1 : 1,000 anti-*α*-actin smooth muscle antibody [1A4] fluorescein isothiocyanate conjugated (FITC) (GTX72521, GeneTex Inc., San Antonio, Texas, USA) for 20 minutes at 4°C. Between every incubation, ASMC were washed with FACS buffer (in mM: 140 NaCl, 2.1 KCl, 8.1 Na_2_HPO_4_ (S3264, Sigma-Aldrich Inc., St. Louis, Missouri, USA), 1.5 KH_2_PO_4_, 0.5% bovine serum albumin (BAH64, Equitech-Bio Inc., Kerrville, Texas, USA), pH = 7.4) and centrifuged for 2 min (1500 rpm at 4°C). ASMC were resuspended in FACS buffer and analyzed for two-color immunofluorescence by flow cytometry (Fluorescent Activated Cell Sorter (FACS) Calibur, Becton Dickinson, Franklin Lakes, NJ, USA). Cells with *α*-actin smooth muscle high expression were selected from a side scatter versus *α*-actin smooth muscle/FITC dot plot. From this gated region, cells expressing *α*-actin smooth muscle/FITC and SHR/Alexa fluor 647 were selected. At least 10,000 events in the *α*-actin smooth muscle high region were analyzed. Data analysis was performed using FlowJo software version 8.7.

### 2.7. Statistical Analysis

Statistical analysis was achieved by one-way analysis of variance (ANOVA) and Bonferroni test for multiple comparisons. A 95% confidence limit and significance was set at *p* < 0.05. Probability values were calculated by Prism 5.0 software (GraphPad, California, USA). All the statistical calculations were made in comparison to control (vehicle) group.

## 3. Results and Discussion

A relationship between the exogenous steroid hormone treatment and the levels of sex hormone receptors in airway smooth muscle in both female and male rats has been clearly detected. For the sake of clarity, in this section ASM is referred to the levels of receptors by Western blot in enzymatically dissociated cells from rat airways, and ASMC to specific airway smooth muscle cells displaying the double staining for *α*-actin smooth muscle and sex hormone receptors as detected by flow cytometry.

### 3.1. ER*α*

**Figure 1:**
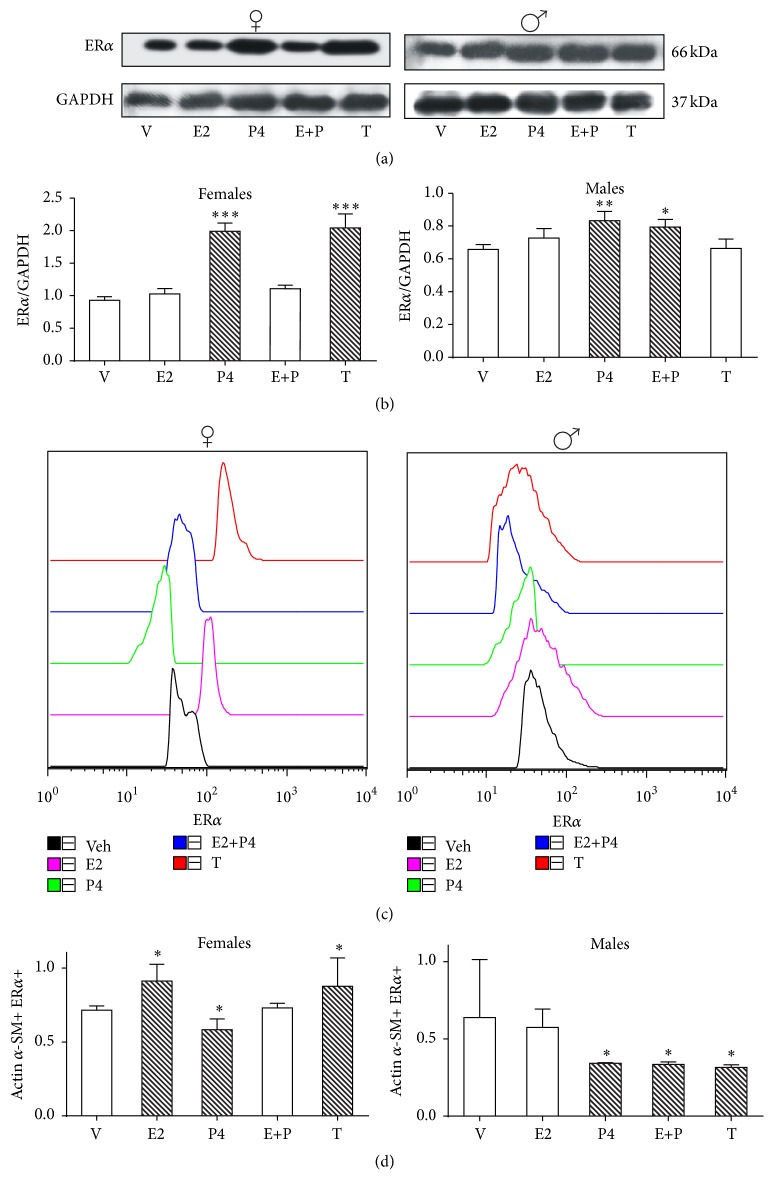
Regulation of ER*α* protein content by sex hormones in airway smooth muscle (ASM) and airway smooth muscle cells (ASMC). ER*α* protein content in ASM and ASMC of gonadectomized female and male rats treated with vehicle (v, corn oil), 17*β*-estradiol (E2), progesterone (P4), 17*β*-estradiol + progesterone (E + P), and testosterone propionate (T). (a) A representative assay of four Western blot experiments is shown. The protein antibody complex was detected by chemiluminescence. Blots were stripped and reprobed with GAPDH antibody to correct differences in the amount of total loaded protein. (b) Proteins detected by Western blot were quantified by densitometric analysis and corrected by GAPDH protein content. Results are expressed as mean ± SD, *n* = 4. ^*∗*^ *p* < 0.05 compared with vehicle treatment, ^*∗∗*^ *p* < 0.01 compared with vehicle treatment, and ^*∗∗∗*^ *p* < 0.001 compared with vehicle treatment. (c) Flow cytometry histogram plots are based on forward scatter (FSC) and side scatter (SSC) gating for ASMC. This was validated using actin *α*-smooth muscle and ER*α* staining. A representative example of the gating strategy is shown. (d) Geometric mean fluorescence intensity (gMFI) for ER*α* expression on ASMC. Results are expressed as mean ± SD, *n* = 4. ^*∗*^ *p* < 0.05 compared with vehicle treatment.

In ASM of female rats, the levels of ER*α* were increased by P4 and T treatments, whereas E2 treatment had no effect on the levels of ER*α* when compared to vehicle. However, E2 blocked the P4 effect on ER*α* expression (Figures 1(a) and 1(b)). Regarding male rats, ASM ER*α* content was upregulated by P4 and E + P (*p* < 0.01 and 0.05, resp.). E2 or T treatments had no effect on ER*α* levels (Figures 1(a) and 1(b)). In brief, ER*α* levels were upregulated by P4 in both sexes, whereas it was upregulated by T in female rats and had no change in males. These results suggest that, in ASM, P4 regulates ER*α* levels in males and females in a similar way. However, T treatment regulation might be influenced by other variables depending on sex; for example, AR is highly expressed in males at protein level while in females it is undetectable in ASM (Figures 4(a) and 4(b)).

Regarding ASMC, we found that T increased ER*α* content in female rats but decreased the content of this receptor in male rats (Figures 1(c) and 1(d)). In female and male mice cerebral cortex T has been shown to reduce ER*α* level [20]. However, the difference between this finding and ours might be due to tissue specific factors that regulate ER*α* levels, such as epidermal factor concentration or protein kinase C (PKC) activation pathways [21, 22].

In female rats, E2 and P4 displayed an opposite effect; that is, E2 upregulated whereas P4 downregulated ER*α* levels. The simultaneous treatment with P4 and E2 had no effect on ER*α* levels in comparison to vehicle, mimicking an allosteric antagonism.

In male rats, P4 downregulated ER*α* levels whereas E2 treatment had no effect. P4 concomitant to E2 treatment downregulated the receptor levels, which might indicate that P4 plays a key role in ER*α* downregulation (*p* < 0.05 compared to vehicle in all cases) (Figures 1(c) and 1(d)).

Townsend et al. [23] showed the presence of ER*α* and ER*β* in human ASMC isolated from adult women bronchi. However, this study did not pursue external hormone treatment, as the authors mention they did not select patients by age, hormone status, or airway disease. In methodological terms, Townsend et al. [23] based their observations on Western blot of epithelium denuded bronchial smooth muscle, with no use of alpha-actin to confirm that the sample to obtain proteins for Western blot was exclusively ASMC. In our experience, we refer to ASM as epithelium denuded smooth muscle where fibroblasts, macrophages, mast cells, and lymphocytes can be found.

### 3.2. ER*β*

**Figure 2:**
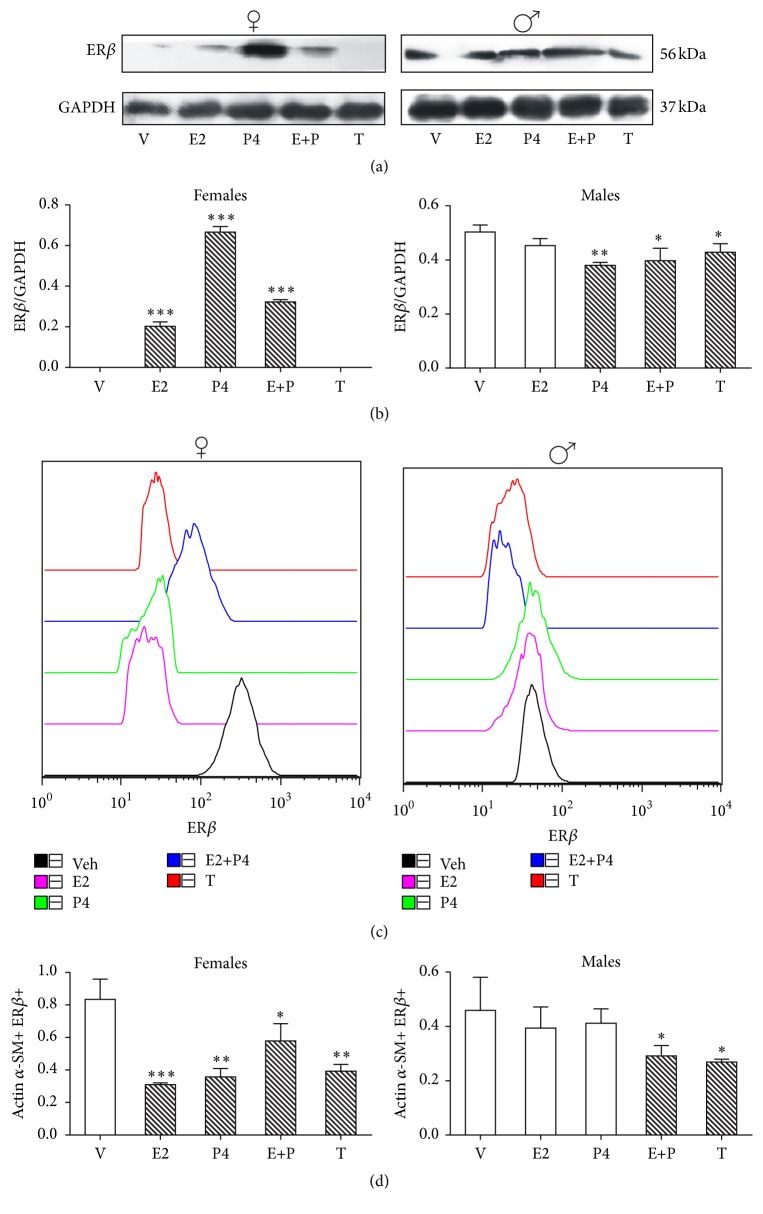
Regulation of ER*β* protein content by sex hormones in airway smooth muscle (ASM) and airway smooth muscle cells (ASMC). ER*β* protein content in ASM and ASMC of gonadectomized female and male rats were treated as described in Figure 1 legend. (a) A representative assay of four Western blot experiments is shown (details are described in Figure 1 legend). (b) Proteins detected by Western blot were quantified by densitometric analysis and corrected by GAPDH protein content. Results are expressed as mean ± SD, *n* = 4. ^*∗*^ *p* < 0.05 compared with vehicle treatment, ^*∗∗*^ *p* < 0.01 compared with vehicle treatment, and ^*∗∗∗*^ *p* < 0.001 compared with vehicle treatment. (c) Flow cytometry histogram plots are based on forward scatter (FSC) and side scatter (SSC) gating for ASMC. This was validated using actin *α*-smooth muscle and ER*β* staining. A representative example of the gating strategy is shown. (d) Geometric mean fluorescence intensity (gMFI) for ER*β* expression on ASMC. Results are expressed as mean ± SD, *n* = 4. ^*∗*^ *p* < 0.05 compared with vehicle treatment. Results are expressed as mean ± SD, *n* = 4. ^*∗*^ *p* < 0.05 compared with vehicle treatment, ^*∗∗*^ *p* < 0.01 compared with vehicle treatment, and ^*∗∗∗*^ *p* < 0.001 compared with vehicle treatment.

ER*β* levels were upregulated in female rats by E2, P4, and E + P treatments in ASM, but not by T when compared with vehicle treatment (*p* < 0.001) (Figures 2(a) and 2(b)). On the contrary, in male rats, P4 (*p* < 0.01) and T (*p* < 0.05) downregulated ER*β* (Figures 2(a) and 2(b)).

The four sex hormonal treatments were able to downregulate ER*β* in ASMC from female rats (E2: *p* < 0.001; P4 and T: *p* < 0.01; E + P: *p* < 0.05). In male rats, only E + P and T downregulated ER*β* (*p* < 0.05) (Figures 2(c) and 2(d)).

It has been reported that ER*β* is downregulated by E2 in the lung of ovariectomized female rats treated with E2 during 48 h [10]. In male rats, E2 and P4 decrease slightly but not significantly ER*β* content in ASMC. This finding might be explained whether a synergistic effect is displayed when both hormones are administered concomitantly with a consequent decrease of the receptor content.

Catley et al. [15] have shown the presence of ER*β* mRNA levels in whole rat lung and airway epithelial cells, macrophages, monocytes, and cultured human airway smooth muscle cells (HASMC) from both sexes. The selection of tissue from only one sex and the certainty to work only with clearly defined smooth muscle cells are crucial for inferences regarding sex regulation and sex hormone receptors. Regarding T effect, this hormone reduces ER*β* levels in cerebral cortex from female and male mice [24].

### 3.3. PR

**Figure 3:**
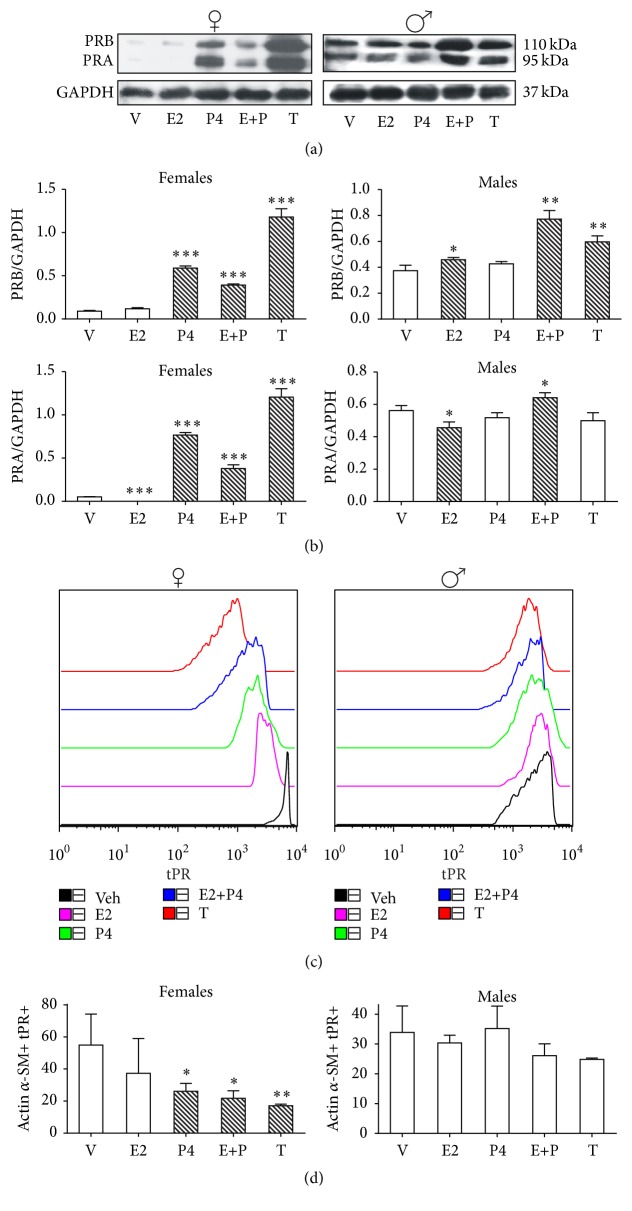
Regulation of PR isoforms protein content by sex hormones in airway smooth muscle (ASM) and airway smooth muscle cells (ASMC). PR isoforms protein content in ASM and total PR (tPR) in ASMC of gonadectomized female and male rats were treated as described in Figure 1 legend. (a) A representative assay of four Western blot experiments is shown (details are described in Figure 1 legend). (b) Proteins detected by Western blot were quantified by densitometric analysis and corrected by GAPDH protein content. Results are expressed as mean ± SD, *n* = 4. ^*∗*^ *p* < 0.05 compared with vehicle treatment, ^*∗∗*^ *p* < 0.01 compared with vehicle treatment, and ^*∗∗∗*^ *p* < 0.001 compared with vehicle treatment. (c) Flow cytometry histogram plots are based on forward scatter (FSC) and side scatter (SSC) gating for ASMC. This was validated using actin *α*-smooth muscle and both isoforms of PR (tPR) staining. A representative example of the gating strategy is shown. (d) Geometric mean fluorescence intensity (gMFI) for tPR expression on ASMC. Results are expressed as mean ± SD, *n* = 4. ^*∗*^ *p* < 0.05 compared with vehicle treatment. Results are expressed as mean ± SD, *n* = 4. ^*∗*^ *p* < 0.05 compared with vehicle treatment and ^*∗∗*^ *p* < 0.01 compared with vehicle treatment.

PR-B in ASM from male rats was upregulated by E2 (*p* < 0.05), E + P (*p* < 0.01), and T (*p* < 0.01) (Figures 3(a) and 3(b)). In different tissues and cell lines PR-B is upregulated by E2. González-Arenas et al. [10] found PRA and PRB protein content upregulation by E2 in whole homogenized lung from female rats. As we have mentioned before, whole homogenized lung is composed by numerous cell lineages. This upregulation by E2 is mediated by estrogen response elements located in the PR promoter [25].

In this work, interestingly E2 showed a statistically significant downregulation on PR-A in ASM from both female and male rats (*p* < 0.001 and *p* < 0.05, resp.) (Figures 3(a) and 3(b)). PRA and PRB were similarly upregulated by P4 and T in ASM from female rats (*p* < 0.001).

Regarding ASMC, in the case of female rats, P4 (*p* < 0.05) and T (*p* < 0.01) downregulated total PR content (tPR). It has been reported that P4 downregulates its own receptor in different tissues and cell lines [10, 26, 27]. This downregulation is induced by ligand-dependent PR phosphorylation resulting in its ubiquitination that marks receptor for degradation by the proteasome 26S [27, 28]. No sex hormone treatment had effect on tPR in male rats. tPR levels have been located in whole lungs as shown by González-Arenas et al. [10]; likely this finding corresponds to other lung cell types different from smooth muscle.

### 3.4. AR

**Figure 4:**
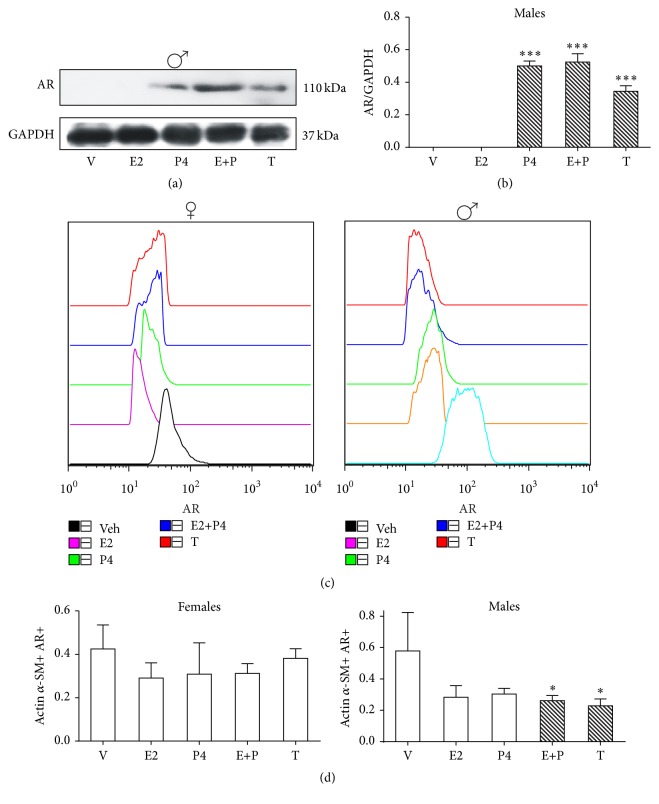
Regulation of AR protein content by sex hormones in airway smooth muscle (ASM) and airway smooth muscle cells (ASMC). AR protein content in ASM and ASMC of gonadectomized female and male rats were treated as described in Figure 1 legend. (a) A representative assay of four Western blot experiments is shown (details are described in Figure 1 legend). (b) Proteins detected by Western blot were quantified by densitometric analysis and corrected by GAPDH protein content. Results are expressed as mean ± SD, *n* = 4. ^*∗∗∗*^ *p* < 0.001 compared with vehicle treatment. (c) Flow cytometry histogram plots are based on forward scatter (FSC) and side scatter (SSC) gating for ASMC. This was validated using actin *α*-smooth muscle and AR staining. A representative example of the gating strategy is shown. (d) Geometric mean fluorescence intensity (gMFI) for AR expression on ASMC. Results are expressed as mean ± SD, *n* = 4. ^*∗*^ *p* < 0.05 compared with vehicle treatment. Results are expressed as mean ± SD, *n* = 4. ^*∗*^ *p* < 0.05 compared with vehicle treatment.

No expression of AR at protein level in females was detected in ASM (Figure 4(a)). However, when ASMC were analyzed with the expression of *α*-actin smooth muscle by flow cytometry, the levels of AR were detected but no statistical difference was found with any of the four different hormonal treatments administered when compared with vehicle (Figure 4(d)). Differences in AR levels between sexes in lung have been reported. Boucher et al. [29] reported that AR expression in lung of female rats is lower when compared with males even if they were castrated.

In male rats, AR levels at protein level in ASM were upregulated by P4 and T treatments (*p* < 0.001) (Figures 4(a) and 4(b)). This result is consistent with a report where testosterone treatment increased AR protein levels in lung cells of castrated male mice [29].

In ASMC, E + P and T downregulated AR levels (*p* < 0.05) (Figures 4(c) and 4(d)).

As in the case of ER*β* content, E2 and P4 decreased slightly but not significantly AR content. It might be likely that the simultaneous treatment with both hormones acts in a synergistic way by decreasing receptor content.

In comparison to E2 and P4, there are few reports on the occurrence of AR in airways. Kimura et al. [30] have reported the expression of AR in epithelial cell nucleus from airway mice. Likewise, AR have been found in lung at early development, bronchial epithelium, and type II pneumocytes [29, 31, 32]. But none of these studies researched particularly AR in ASMC or their hormone regulation.

The steroid receptors studied in this work displayed differences in protein content between Western blot detection and flow cytometry. In this regard, there are two points that should be considered. First, flow cytometry is a highly sensitive technique allowing the observation of suitable changes that cannot be detected by Western blot. Second, different cell types can be found in ASM, the proper smooth muscle cells, fibroblasts, and stem cells [33]. All these cell types express steroid receptors [34]. It is likely that regulation of these nuclear receptors is different in each cellular lineage. This might explain differences in ER, PR, and AR levels between ASM and ASMC results, hence, the importance of studying cell populations separately to determine cell specific changes in ASM.

To our knowledge, this is the first report of PR regulation by testosterone and AR regulation by progesterone in ASMC. Our results demonstrate that the lung expression of ER*α* and ER*β* is upregulated in females by P4, ER*β* by E2, and ER*α* by T, suggesting that sex hormones might induce a protective environment to inflammation and airway contraction in lung cells. Nevertheless, these findings disagree with the evidence that suggests that women have more severe asthma than men. A likely explanation of this phenomenon is that the expression of not only ER*β* but also PR and AR in female airway smooth muscle is downregulated by sex hormones. ER*α* was the unique receptor upregulated by its own ligand, E2, and also by T. This upregulation of ER*α* is probably reducing the negative effects of the downregulation exerted by other hormone receptors.

Paradoxically, we have observed that AR receptor was also downregulated by its ligand, testosterone, and the other sex hormones. This is a controversial finding in view that testosterone and estrogen contribute to regulate smooth muscle tone [9]. A plausible explanation of our findings might be associated with the endocrinology regulation of sex hormones. Female hormones present cyclic level changes, but male sex hormones do not. In this context, estrogen and progesterone are “withdrawn” in females, for example, during menstrual cycles, worsening symptoms in disease as asthma because, as it has been observed in our results, the hormone receptors are downregulated.

Potvin et al. have determined the role of the nuclear progesterone receptor by using wild-type (WT) and PR knock-out (PRKO) mice at postnatal days 1, 4, and 10. They measured the hypoxic ventilatory response (14 and 12% O_2_, 20 min each) and apnoea frequency in both male and female mice by using whole-body plethysmography. They conclude that PR is a key contributor to the hypoxic ventilatory response in newborn mice, but PR deletion does not increase the frequency of apnoea during normoxia or hypoxia [35].

Various studies indicate that sex hormones, in particular estrogens, have protective effects against bronchoobstruction and inflammation. For example, stimulation of ER*α* and *β* receptors in human bronchial epithelial cells with estrogen induced nitric oxide production that results in bronchodilatation [36]. In addition, G-protein-coupled estrogen receptor has been associated with immunoregulatory functions by increasing IL-10 production in airways [37]. In a recent study, estrogen-treated mice had higher expression of the anti-inflammatory mediator secretory leukoprotease inhibitor [38]. In relation to smooth muscle, estrogens stimulation has an important role relaxing this tissue [23].

We are aware that our findings have implications in pulmonary processes such as inflammation, remodeling, and hyperresponsiveness, as hallmarks of diseases where a sex hormone role has been proposed, such as asthma, pulmonary fibrosis, and chronic obstructive pulmonary disease (COPD). Sex hormone regulation of sex hormone receptors in smooth muscle is relevant as well in interplay and cross talk among all the cell types involved in lung physiological processes either in health or in disease.

## 4. Conclusions

In conclusion, we have shown the occurrence of the three sex hormone receptors specifically in ASMC and their regulation by the sex hormones, as well as in ASM, in female and male rats. The regulation of steroid receptors by steroid hormones depends on the cellular lineage and the hormonal environment.
